# Gut microbial taxa as potential predictive biomarkers for acute coronary syndrome and post-STEMI cardiovascular events

**DOI:** 10.1038/s41598-020-59235-5

**Published:** 2020-02-14

**Authors:** Jing Gao, Kun-Tao Yan, Ji-Xiang Wang, Jing Dou, Jie Wang, Min Ren, Jing Ma, Xu Zhang, Yin Liu

**Affiliations:** 1 Cardiovascular Institute, Tianjin Chest Hospital, No.261 Tai er zhuang Road, Jinnan District Tianjin, 300222 P. R. China; 2 TEDA International Cardiovascular Hospital, No.61,Third Street, Economic and Technological District Tianjin, 300457 P. R. China; 3 Department of Cardiology, Tianjin Chest Hospital, No.261 Tai er zhuang Road, Jinnan District Tianjin, 300222 P. R. China; 4 Tianjin Medical University, No.22 Qi xiang tai Road, Heping District Tianjin, 300070 P.R. China

**Keywords:** Cardiovascular diseases, Acute coronary syndromes, Microbiology, Cardiology

## Abstract

Plasma trimethylamine N-oxide (TMAO) is associated with coronary atherosclerotic plaque and cardiovascular disease risk, but associations between gut microbes in acute coronary syndrome (ACS) and post-ST-segment elevation myocardial infarction (post-STEMI) events are unknown. We investigated associations between gut microbial taxa and systemic TMAO levels and the possible TMAO contribution to incident post-STEMI cardiovascular events. Patients and Methods. A total of 60 patients, including 30 with unstable angina pectoris (UAP), 30 post-STEMI and 30 healthy controls, were enrolled from June to November 2017. Metagenomic sequencing was performed and TMAO and IL-6 were detected. Results. Minimal discriminators of gut microbial taxa (top 40) distinguished ACS patients from controls. Serum TMAO levels were positively associated with increased abundance of *Aerococcaceae, Ruminococcaceae_UCG.005, Ruminococcaceae_UCC.014* and *X. Eubacterium_fissicatena*, and decreased abundance of *Lachnospiraceae_FCS020* (P < 0.05). Elevated serum TMAO levels correlated independently with ACS (P < 0.05). Risk stratification for incident major adverse cardiovascular events (MACE) improved at one year in patients with serum TMAO levels ≦2.19 µM. Serum interleukin-6 levels were not significantly increased in patients with ACS and post-STEMI MACE. Conclusions. ACS and incident post-STEMI MACE may be associated with the gut bacteria choline metabolite TMAO. The specific gut microbial taxa identified in association with serum TMAO levels may be potential predictive biomarkers for accurate diagnosis of ACS onset.

## Introduction

Myocardial infarction (MI) is a frequent cause of heart failure and cardiac death worldwide^1^, accounting for about 18 million deaths annually, or about 30% of all deaths globally^2,3^. Strategies for preventing recurrent MI include suppression of inflammatory response, lipid level reduction, and regulation of multiple suppressive pathways to protect against adverse remodeling^4^. However, although mechanical reperfusion by percutaneous coronary intervention has reduced the acute mortality rates of MI, the incidence of post-MI cardiovascular events is still high and still predicts increased mortality^4^. Dating and predicting these events constitutes a clinical challenge.

Since awareness increased markedly regarding the involvement of gut microbes in the development of numerous cardiometabolic disease^5–7^. Recent reports have highlighted the involvement of gut microbes in the pathogenesis of both related adverse thrombotic events and atherosclerotic heart disease^8–12^. Specific trimethylamine (TMA)-containing dietary nutrients, such as carnitine, choline, and phosphatidylcholine, is as a source of carbon fuel used by gut microbes. The waste compound TMA is carried to the liver via portal circulation, where host hepatic flavin monooxygenase (FMO)-dependent conversion metabolizes it into trimethylamine N-oxide (TMAO)^8–13^. Numerous studies have revealed a correlation between cardiovascular risk and serum TMAO levels in both stable cohorts and animal models^8–10,12,14–16^. Also, recent studies involving genetic manipulation of FMO3, the major FMO responsible to convert TMA into TMAO^13^, have confirmed that this meta-microbial pathway is involved in both the regulation of sterol and total cholesterol metabolism and the development of atherosclerotic plaque^17–19^. Moreover, gut microbes have been identified to have an obligatory role in TMAO generation in humans^9,10^. The association between serum TMAO level and the extent of both the coronary atherosclerotic plaque burden and cardiovascular disease (CVD) risk has been demonstrated in multiple clinical studies^8,9,15,20,21^.

However, associations between serum TMAO level and the risk of incident cardiovascular events in patients with ST-segment elevation myocardial infarction (STEMI) has not yet been examined. This association is particularly relevant given that TMAO is shown in human and animal studies to link with altering stimulus-dependent calcium signalling, platelets, enhancing thrombosis potential, and fostering platelet hyperreactivity *in vivo*^12^. Platelet hyperreactivity is a risk factor for the occurrence of cardiovascular events. TMAO was also shown in animal models to enhance up-regulation of adhesion proteins, inducing aortic endothelial cell activation, and vascular inflammation^9,10^. In addition, MI elevated gut mucosa permeability and drives intestinal barrier failure, resulting in the translocation of microbial products and gut microbes into the systemic circulation, which induces additional immune inflammation and increases the risk of cardiovascular events after MI^16^. Elevated serum TMAO level among stable subjects undergoing elective diagnostic cardiac evaluations predict the risk of incident thrombotic events^12^. The clinical importance of serum TMAO levels on cardiovascular events in patients was also demonstrated in a study finding that microbiota-derived TMAO predicted post-SETEMI cardiovascular risk^22^. Nevertheless, the involvement of specific gut microbes and TMAO in acute coronary syndrome (ACS) and incident post-STEMI cardiovascular events has not yet been reported. Therefore, the purpose of this study was to explore associations between gut microbial taxa and systemic TMAO levels, and to investigate the contribution of the gut microbial metabolite TMAO to incident cardiovascular events after STEMI.

## Patients and Methods

### Study design and sample

This prospective study enrolled a convenience sample of 60 ACS patients, including 30 STEMI and 30 unstable angina pectoris (UAP) patients treated in the cardiac/coronary care unit (CCU) of Tianjin Chest Hospital from June 2017 to November 2017, and 30 healthy volunteers from the hospital health examination center during the same period. Convenience sample is defined as a non-probability/non-random sample of subjects nearest and most available to participate in this study. Inclusion criteria were diagnoses of UAP or STEMI, defined as follows:

#### STEMI diagnostic criteria

cardiac troponin (cTn) I/T > the upper limit of the normal reference value or creatine kinase isoenzyme > the upper limit of the normal reference value; electrocardiogram (ECG) showed ST segment elevation on 2 or more adjacent leads; and one or more of the following: persistent ischemic chest pain, abnormal segmental wall motion upon ECG, and abnormal coronary angiography.

#### UAP diagnostic criteria

cTnI/T negative; ischemic chest pain; and either transient ST-segment depression / low-level T-wave or inverted, rare ST-segment elevation upon ECG.

Exclusion criteria were: history of organic digestive system or digestive tract surgery; history of stroke, hypertension, diabetes, kidney disease or respiratory diseases; history of smoking or alcohol abuse; infection within one month of the study or the use of a probiotic, antacid, antibiotic, or antibiotic preparation.

### Ethical considerations

The study protocol was approved by the Ethics Committee of Tianjin Chest Hospital (No. 2018KY-010-01) and all subjects provided signed informed consent to participate. All procedures performed in studies involving human participants were in accordance with the ethical standards of the Helsinki declaration and its later amendments or comparable ethical standards.

### Specimens

Fasting blood specimens and fresh stool specimens were collected at admission. Blood specimens were centrifuged at 4 °C, 3000 rpm for 10 minutes, and the supernatant (serum, plasma) was frozen and stored at −80 °C for testing. The first morning fresh fecal specimens (>300 mg) were collected from the STEMI group, UAP group and the healthy control group in a picking box, sealed and transported to the sample bank at 4 °C. Fecal specimens (300 mg) were placed into a sterile externally-circulated cryotube, and then sealed and placed in a refrigerator at −156 °C for storage.

### DNA extraction and library construction

Genomic DNA was extracted from specimens using the cetyl trimethylammonium bromide method. Genomic DNA was diluted to 1 ng/μl and used as a template, the 16 S V4 region was amplified as a target. The primer sequences were 515 F (5′-GTGCCAGCMGCCGCGGTAA-3′) and 806 R (5′-GGACT CHVGGGTWTCTAAT-3′). Polymerase chain reaction (PCR) was performed using Phusion High-Fidelity PCR Master Mix. The PCR product was purified by Thermo Scientific kit (Thermo Fisher Scientific, Waltham, MA, USA). The DNA library was constructed using Thermo Fisher Ion Plus Fragment Library Kit 48 rxns library, and subjected to Qubit quantification and library testing, then sequenced using Thermo Fisher Life Ion S5TM or Ion S5TMXL.

### Sequencing data processing, operational taxonomic units clustering, and species annotation

Low-quality partial shearing of reads was accomplished using Cutadapt (V1.9.1, http://cutadapt.readthedocs.io/en/stable/)^23^, with Barcode and preliminary control of primer sequences truncated to obtain raw data (Raw reads). The Reads sequence was compared with the Gold database (http://drive5.com/uchime/uchime_download.html) to detect the chimeric sequences^24^, which were then removed to obtain the final valid data (Clean Reads)^25^. UPARSE software (UPARSE Version 7.0.1001, http://drive5.com/uparse/) was used to cluster all valid bases of all samples into Operational Taxonomic Units (OTUs)^26^, and all sequences were subjected to OTU partitioning and biological information according to specified similarity (default 97%) analysis. The Mothur method (Mothur Version 1.18.0) and SILVA’s SSUrRNA (https://www.arb-silva.de/) database were used to analyze the representative sequences, and the MUSCLE software (MUSCLE Version 3.8.31) was used for OTU sequence analysis; Qiime software (Version 1.9.1) was used to analyze α and β diversity and the difference in inter-group species.

### Detection of TMAO and IL-6

A Hypersil HILIC hydrophilic column (specification 150 × 2.1 mm, 1.7 μm, ThermoFisher Scientific, Waltham, MA, USA) was constructed as follows: mobile phase A: water (5 mM ammonium acetate); mobile phase B: acetonitrile. Conditions for the gradient elution using gradient program were: 0–5 min, 50% A; 5–10 min, 50% A-80%, 10–15 min, 80% A, total run time 15 min; flow rate: 0.3 mL/min; amount: 3 μL; column temperature: 30 °C. The electrospray ion source was in positive ion mode with high purity nitrogen (purity 99.999%) as dry gas, drying gas temperature 325 °C, flow rate 10 L/min. The scanning method was in multiple reaction monitoring mode, quantitative ion pair: TMAO m/z 76 → 58, d9-TMAO m/z 85 → 66; qualitative ion: TMAO m/z 42, d9-TMAO m/z 46; and collision energy: TMAO 20 eV, d9-TMAO 25 eV. Analyses were conducted using 1260 Infinity High Performance Liquid Chromatograph (Agilent, Santa Clara, CA, USA) and 6420 Triple Quad Triple Quadrupole Mass Spectrometer (Agilent, Santa Clara, CA, USA). Circulating interleukin-6 (IL-6) levels were measured using commercial ELISA kits (Abcam, Cambridge, MA, USA) according to the manufacturer’s instructions.

### Patient follow-up

All STEMI patients were followed for one year after STEMI onset. The occurrence of a first MACE was regarded as the follow-up endpoint, including cardiac death, non-fatal ischemic stroke, recurrent MI, need for emergency or repeat revascularization, and re-hospitalization for heart failure, as defined above under Study Design and Sample.

### Statistical analysis

Demographic and clinical characteristics, prognostic markers, and microbial taxa are presented as n (%) for sex and mean ± standard deviation (SD) or median with 1st and 3rd quartiles (Q1, Q3) for other numerical data by group. Differences between groups were compared using Fisher’s exact test for sex; two-sample t-test for numerical data with normal distribution; Mann-Whitney U test for numerical data without normal distribution; and two-way ANOVA for numerical data after adjusting for variables that varied significantly by demographic or clinical characteristics. Receiver operating characteristic (ROC) curve analysis was performed to identify one-year major adverse cardiovascular events (MACE) in terms of the identified prognostic markers. A Kaplan-Meier curve was graphed for MACE-free survival time, angina-free survival time, and any event-free survival time of patients by high and low TMAO levels. High and low TMAO levels were classified according to the median value of the control group. A scatter plot of TMAO with associated microbial taxa was constructed with the coefficient of Spearman’s correlation analysis. For post-STEMI patients, the demographic and clinical characteristics, and prognostic markers are presented as n (%) for sex or median (Q1, Q3) for other numerical data of one-year recurrence of MACE; differences between patients with and without MACE were compared using Fisher’s exact test for sex and Mann-Whitney U test for other variables. All statistical assessments were two-tailed and considered significant at P < 0.05. All statistical analyses were carried out using IBM SPSS statistical software version 22 for Windows (IBM Corp., Armonk, NY, USA).

## Results

### Study population

A total of 90 consecutive cardiology patients were enrolled and, after excluding 30 patients according to the exclusion criteria, the remaining 60 were divided into two groups for analysis: an ACS group, including 30 post-STEMI patients and 30 UAP patients; and a control group comprising 30 healthy volunteers. Although the controls were not age- and gender-matched with the ACS group, no evidence of enrollment bias was found.

### Characteristics of study participants

The baseline characteristics of UAP patients, STEMI patients and healthy controls are shown in Table 1. Compared to healthy controls, ACS patients had higher admission levels of systolic and diastolic blood pressure, alanine aminotransferase (ALT), lipoprotein (a), admission glucose, and uric acid; elevated white blood (WBC) count; reduced platelet count; and lower levels of albumin and high-density lipoprotein (P < 0.05). In addition, significant differences were found between groups in HR time, WBC count, and the levels of albumin, ALT, and admission glucose (P < 0.05). (Table 1).

**Table 1:** Comparisons of demographic and clinical characteristics between ACS patients and controls.

	Control	ACS			p-value	p-value
		Total ACS	UAP	STEMI	Control vs. total ACS	UAP vs. STEMI
*n*	*30*	*60*	*30*	*30*		
Sex, males (%)	22 (73.3)	48 (80)	25 (83.3)	23 (76.7)	**0.369**	0.748
Age, year	52.65 ± 8.79	54.92 ± 8.51	56.93 ± 7.15	52.9 ± 9.37	**0.251**	0.066
BMI (kg/m^2^)	24.39 ± 2.86	25.48 ± 2.42	25.34 ± 2.21	25.61 ± 2.64	0.062	0.671
HR (/min)	73.27 ± 8.69	73.22 ± 16.34	66.23 ± 8.05	80.20 ± 19.43	0.985	**0.001**
SBP (mmHg)	123.27 ± 16.18	137.52 ± 20.64	140.67 ± 21.12	134.37 ± 20	**0.001**	0.240
DBP (mmHg)	73.2 ± 8.47	82.37 ± 14.3	82.37 ± 14.39	82.37 ± 14.45	**<0.001**	1.000
WBC (10^9^/L)	6.79 ± 1.25	9.14 ± 3.46	6.85 ± 1.77	11.44 ± 3.21	**<0.001**	**<0.001**
HB (g/L)	138.73 ± 12.42	141.88 ± 19.05	145.63 ± 17.37	138.13 ± 20.18	0.349	0.128
PLT (10^9^/L)	260.2 ± 53.53	226.45 ± 54.73	215.13 ± 47.61	237.77 ± 59.69	**0.007**	0.110
ALB (g/L)	47.67 ± 2.82	42.36 ± 4.49	44.47 ± 3.93	40.25 ± 4.04	**<0.001**	**<0.001**
ALT (U/L)	14.5 (8.9, 23.4)	26.55 (17.30, 52.95)	16.10 (14.9, 26.1)	50.35 (27, 66.4)	**<0.001**	**<0.001**
Lp(a) (nM)	14.55 (6.7, 45.7)	34.2 (9.7, 103.35)	49.55 (16.20, 119)	28.05 (8.5, 81.9)	**0.030**	0.217
TC (mM)	4.71 ± 0.95	4.51 ± 1.23	4.23 ± 0.94	4.80 ± 1.41	0.449	0.070
TG (mM)	1.4 (0.95, 1.97)	1.63 (1.13, 2.30)	1.87 (1.19, 2.57)	1.52 (1.05, 1.99)	0.078	0.352
HDL-C (mM)	1.33 ± 0.35	1.02 ± 0.29	1 ± 0.23	1.04 ± 0.35	**<0.001**	0.616
LDL-C (mM)	3.09 ± 0.79	2.92 ± 1.09	2.77 ± 0.84	3.07 ± 1.3	0.432	0.289
GLU (mM)	5.04 (4.82, 5.28)	5.77 (5.06, 7.39)	5.44 (4.92, 6.07)	6.32 (5.52, 8.08)	**<0.001**	**0.013**
CR (μM)	72.07 ± 17.77	80.47 ± 23.52	84.87 ± 18.7	76.07 ± 27.13	0.088	0.149
UA (μM)	315 ± 76.22	364.32 ± 114.66	364.97 ± 84.97	363.67 ± 139.74	**0.036**	0.965

Data are presented as n (%) for sex; mean ± SD or median (Q1, Q3) for other numerical data.

Differences between two groups were compared using Fisher’s exact test for sex; two-sample t-test for numerical data with normal distribution; Mann-Whitney U test for numerical data without normal distribution.

Bold values indicate statistical significance (p < 0.05).

Abbreviations: ACS, acute coronary syndrome; UAP, unstable angina pectoris; STEMI, ST-segment elevation myocardial infarction; BMI, body mass index; HR, heart rate; SBP, systolic blood pressure; DBP, diastolic blood pressure; WBC, white blood cell; HB, hemoglobin; PLT, platelet; ALB, albumin; ALT, alanine aminotransferase; Lp(a), Lipoprotein(a); TC, total cholesterol; TG, triglycerides; HDL-C, high-density lipoprotein cholesterol; LDL-C, low-density lipoprotein cholesterol; GLU, serum glucose; CR, creatinine; UA, uric acid.

During one-year follow-up of STEMI patients, as shown in Supplementary Table 1, seven patients had MACE, including one cardiovascular death, two recurrent MIs, three repeat revascularizations, and one hospitalization for heart failure. A significant difference was observed between patients with and without MACE in serum tri-glycerol on day 2 of STEMI onset. (Supplementary Table 1).

### Specific gut microbial taxa associated with ACS onset

**Figure 1:**
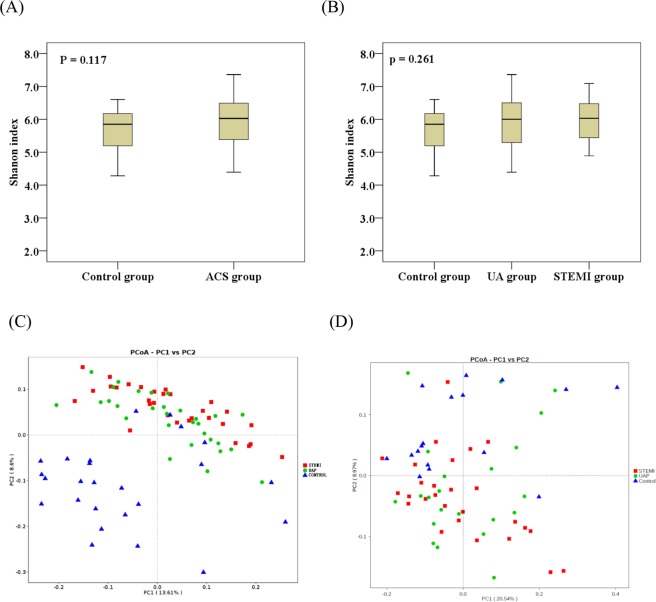
Gut microbial composition in stool of human adults with or without ACS. The microbial abundance and α-diversity (Shannon index) based on the profile of family and genera between (**A**) control and ACS groups and (**B**) control, UAP, and STEMI groups. Principle Coordinate Analysis (PCoA) (weighted UniFrac distance) showed that ACS and control groups were distinguished by total (**C**) or top 40 (**D**) abundant stool microbial taxa (P < 0.05), yet overlapping clustering was observed between UAP and STEMI groups.

An average of 73,754 and 79,955 high-quality sequences were generated per sample for ACS patients and healthy controls, respectively (Supplementary Table 2). No significant differences were found between ACS patients and controls in effective base sequences (P > 0.05). The within-sample (α) diversity (presented as Shannon index) was increased in the ACS group compared with that in the control group (Fig. 1A,B). However, no significant differences were found in the Shannon index between ACS patients and controls (P > 0.05).

Principle Coordinate Analysis (PCoA) (weighted UniFrac distance) showed that the ACS and control groups were distinguished by total (Fig. 1C) or top 40 (Fig. 1D) abundant stool microbial taxa (P < 0.05), and overlapping clustering was observed between UAP and STEMI groups.

**Figure 2:**
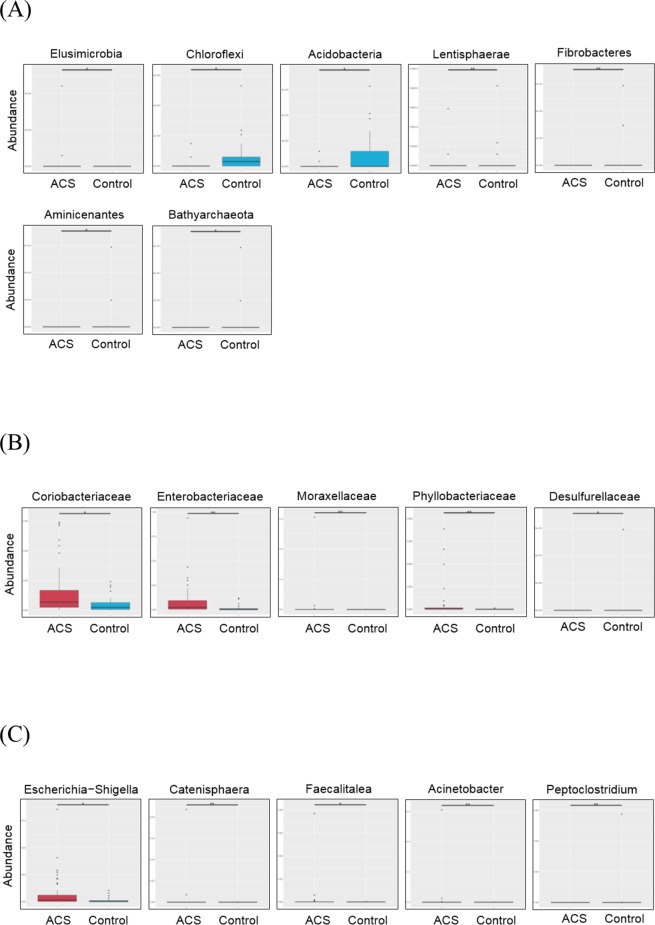
The box plot of abundance for gut microbial taxa in control and ACS groups. The levels of phylum (**A**), family (**B**), and genera (**C**).

At the phylum level (Fig. 2A), the dominant stool microbes in the ACS group were *Elusimicrobia* (adjusted P = 0.012), while the dominant stool microbes in the control group were *Acidobacteria* (P-adj = 0.0018), *Chloroflexi* (P-adj = 0.023), *Lentisphaerae* (Padj = 0.04), *Aminicenantes* (P-adj = 0.025), *Bathyarchaeota* (P-adj = 0.012), and *Fibrobacteres* (P-adj = 0.02). At the family level (Fig. 2B), the ACS group was rich in *Coriobacteriaceae* (P-adj = 0.012), *Enterobacteriaceae* (P-adj = 0.009), *Moraxellaceae* (P-adj = 0.005), and *Phyllobacteriaceae* (P-adj = 0.005), while the control group was rich in *Desulfurellaceae* (P-adj = 0.028). At the genera level (Fig. 2C), the ACS group was rich in *Escherichia-Shigella* (P-adj = 0.027), *Catenisphaera* (P-adj = 0.007), *Faecalitalea* (P-adj = 0.016), and *Acinetobacter* (P-adj = 0.007), while the control group was rich in *Peptoclostridium* (P-adj = 0.007).

**Figure 3:**
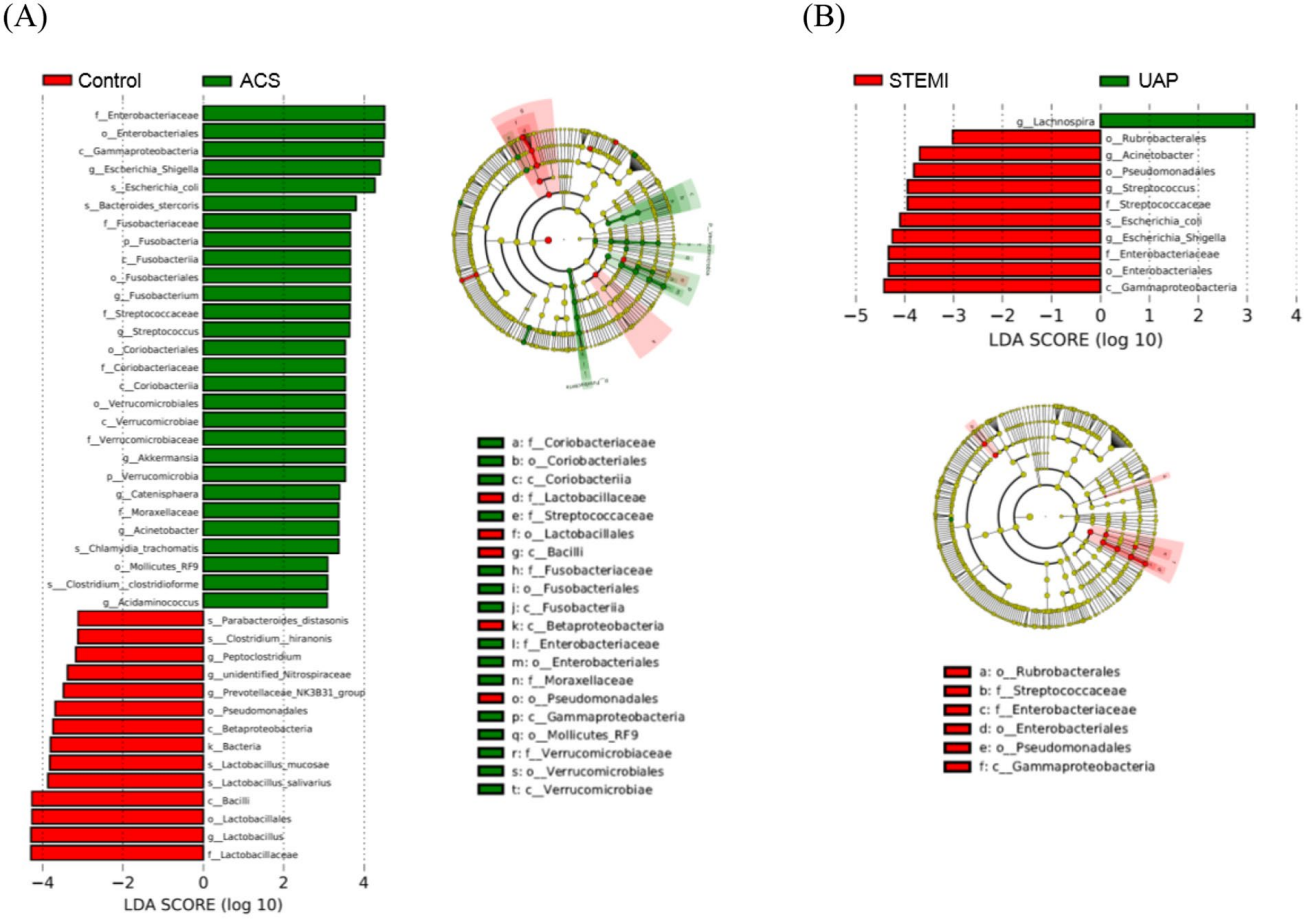
Elevated abundance of gut microbial taxa in human adults with ACS. Relative abundance of the different orders, families, or genera were identified through LEfSe analysis (LEfSe score > 3) as being characteristics of ACS versus control group (**A**) and STEMI versus UAP groups (**B**).

Differences observed in effect-size measures of gut microbiota between control and ACS groups are shown in Fig. 3A. In total, 18 taxa of order, family, or genera were identified through LEfSe analysis (LEfSe score > 3) as being characteristics of ACS versus the control group: significantly increased abundance of 13 families or genera (*Enterobacteriaceae, Fusobacteriaceae, Streptococcaceae, Coriobacteriaceae, Verrucomicrobiaceae, Moraxellaceae, Escherichia-Shigella, Fusobacterium, Streptococcus, Akkermansla, Catenisphaera, Acinetobacter*, and *Acidaminococcus*) and decreased abundance of five family or genera (*Lactobacillaceae, Peptoclostridium, unidentified_Nitrospiraceae, Prevotellaceae_NK3B31_group*, and *Lactobacillus*). In addition, compared to UAP groups, the STEMI group had significantly increased abundance of five families or genera (*Streptococcaceae, Enterobacteriaceae, Acinetobacter, Streptococcus*, and *Escherichia-Shigella*) and decreased abundance of one genera (*Lachnospira*) (Fig. 3B).

### Products of specific gut microbial taxa associated with ACS onset

Results of TMAO levels in blood samples of ACS patients collected on day 2 of symptom onset are shown in Table 2. Compared to the control group, serum TMAO levels were significantly increased in ACS patients but no significant differences were found between STEMI and UAP patients. As shown in Table 2, serum IL-6 levels were not significantly increased in the ACS group compared to the control group. Compared to the UAP groups, serum IL-6 levels were also not significantly increased in the ACS group compared with the control group. (Table 2).

**Table 2:** Comparison of prognostic markers between controls and ACS patients.

	Control	ACS			p-value (p-adj.)	p-value (p-adj.)
		Total ACS	UAP	STEMI	Control vs. Total ACS	UAP vs. STEMI
*n*	*30*	*60*	*30*	*30*		
TMAO(μM)	2.15 ± 0.9	4.33 ± 2.49	4.31 ± 2.8	4.35 ± 2.19	**<0.001** (0.221)	0.954 (**0.004)**
IL-6(pg/ml)	20.64 ± 5.95	34.54 ± 79.68	25.95 ± 18.12	43.12 ± 111.52	0.344 (0.204)	0.409 (0.124)

Data are presented as mean ± SD or median (Q1, Q3) for all numerical data.

Differences between two groups were compared using two-sample t-test for numerical data with normal distribution; Mann-Whitney U test for numerical data without normal distribution; two-way ANOVA after adjusting for variables significantly different in Table 1 (with p < 0.05).

Bold values indicate statistical significance (p < 0.05).

Abbreviations: ACS, acute coronary syndrome; UAP, unstable angina pectoris; STEMI, ST-segment elevation myocardial infarction; TMAO, trimethylamine N-oxide; IL-6, interleukin-6.

Among the detected gut microbes within all groups, five taxa were identified whose abundance was significantly associated with serum TMAO levels: *Aerococcaceae, Ruminococcaceae_UCG.005, Ruminococcaceae_UCC.014, X.Eubacterium_fissicatena*, and *Lachnospiraceae_FCS020*, (P < 0.05) (Table 3). Compared to the control group, the abundance of *Aerococcaceae* was significantly increased in STEMI patients specifically, and the abundance of *Eubacterium_fissicatena* was significantly increased in ACS patients (Table 4). Alterative microbial taxa whose abundance was significantly reduced in ACS or STEMI groups, such as *Lachnospiraceae_FCS020*, were associated with lower TMAO levels.

**Table 3:** Associations between detected gut microbial taxa and serum TMAO levels within all groups.

Gut microbial taxa	Correlation Coefficient	p-value
Ruminococcaceae_UCG.005	0.541	0.004*
X.Eubacterium_fissicatena	0.548	0.003*
Lachnospiraceae_FCS020_group	−0.700	**<**0.001*
Ruminococcaceae_UCG.014	0.549	0.003*
Aerococcaceae	0.632	**<**0.001*

^*^Indicates statistical significance (p < 0.05).

Abbreviations: TMAO, trimethylamine N-oxide.

**Table 4:** Differences in prognostic markers and TMAO-related gut microbial taxa between ACS and control groups.

Microbial taxa	Control	ACS			p-value	p-value	p-value
		Total ACS	UAP	STEMI	Control vs. total ACS	Control vs. STEMI	UAP vs. STEMI
Ruminococcaceae_UCG.005	0.17 ± 0.11	0.16 ± 0.13	0.13 ± 0.10	0.175 ± 0.150	0.590	1.000	0.595
X. Eubacterium_fissicatena_group	0.02 ± 0.05 × 10^−3^	0.03 ± 0.05 × 10^−3^	0.04 ± 0.06 × 10^−3^	0.02 ± 0.04 × 10^−3^	**0.037**	0.058	0.114
Lachnospiraceae_FCS020_group	0.17 ± 0.11 × 10^−3^	0.10 ± 0.15 × 10^−3^	0.10 ± 0.18 × 10^−3^	0.09 ± 0.13 × 10^−3^	**0.005**	**0.015**	0.624
Ruminococcaceae_UCC.014	0.0071 ± 0.0192	0.0172 ± 0.0536	0.0094 ± 0.0228	0.025 ± 0.070	0.139	0.141	0.662
Aerococcaceae	0.29 ± 1.19 × 10^−5^	1.57 ± 0.59 × 10^−5^	0.19 ± 1.07 × 10^−5^	2.94 ± 8.07 × 10^−5^	0.308	**0.014**	**0.022**

Data are presented as mean ± SD by group.

Differences between twogroups were compared using Mann-Whitney U test for numerical data without normal distribution.

Bold values indicate statistical significance (p < 0.05).

Abbreviations: TMAO, trimethylamine N-oxide; ACS, acute coronary syndrome; UAP, unstable angina pectoris; STEMI, ST-segment elevation myocardial infarction.

**Figure 4:**
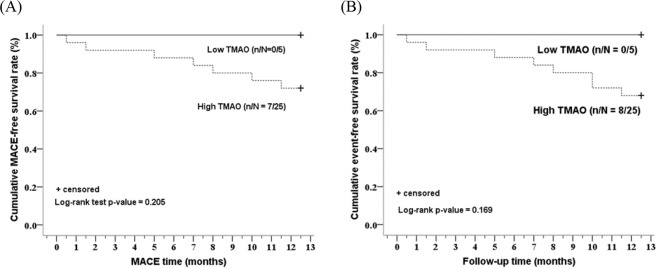
Comparison of 1-year cardiovascular events of post-STEMI between patients with low and high serum TMAO levels. 1-year cardiovascular events included 1-year MACE-free (**A**) or total event-free survival rate (**B**). Patients were stratified according to the median value = 2.19 for TMAO in controls. n indicates MACE or total events, N indicates total number of patients.

The results of Kaplan-Meier curve analyses revealed that an optimal cut-off value of TMAO could identify subjects at higher risk for post-STEMI MACE (Fig. 4). After 1-year follow up, MACE occurred in 7 (23%) patients and the total number of events occurred in 8 (27%) STEMI patients. Kaplan-Meier and ROC curve analysis **(**Supplementary Table 3**)** showed that higher TMAO levels led to more efficient risk stratification in STEMI patients, which predicted the decrease of MACE-free or total event-free survival rates. ROC curve analysis shows only a trend to significance for the prediction of MACE by the TMAO levels. However, the association between TMAO levels and these cardiovascular events was not significant (MACE-free survival: P = 0.205; total event-free survival rate: P = 0.169) (Fig. 4). Kaplan-Meier curve survival plot analysis also showed that gut microbial taxa, including *Aerococcaceae*, *Eubacterium_fissicatena* and *Lachnospiraceae_FCS020*, were not associated with the occurrence of MACE in STEMI patients (Supplementary Fig. 1).

## Discussion

Results of the present study demonstrated that the gut microbial taxa of ACS patients are clearly different from those of subjects without ACS. The difference may represent a shift in the community composition of gut microbes that correlates with serum levels of the gut microbiota-related metabolite TMAO and the occurrence of post-STEMI cardiovascular events. Because the TMAO baseline increases in STEMI with MACE lacked significance, a trend is suggested but further study is needed to confirm a definitive correlation. Nevertheless, minimal discriminators of top 40 microbial taxa were readily able to distinguish ACS patients from healthy controls. The specific gut microbial taxa identified and the association with serum TMAO levels may have the potential to be predictive biomarkers for the accurate diagnosis of ACS onset.

In the present study, we noted overlapping clustering between UAP and STEMI groups, suggesting that gut microbial taxa are associated with the occurrence of ACS. In a previous study, distinct (non-overlapping) clusters were observed between healthy controls and ACS patients by PCoA based on the total blood microbial taxa^12^, suggesting the presence of different microbial community structures between controls and ACS patients. The present study expands on these findings and shows that specific gut microbes, via TMAO generation, are directly associated with the occurrence of ACS. Specifically, the stool microbiome of STEMI patients had a higher abundance of *Aerococcaceae* and *Eubacterium* and a lower abundance of the beneficial microbe family *Lachnospiraceae*, results that were associated with serum TMAO levels. In another study, compared with controls, patients with type 2 diabetic erectile dysfunction who had increased serum TMAO levels also had a corresponding increase in relative abundance of *Aerococcus* (opportunistic pathogens) and a decrease in *Eubacterium*^27^. In mice, *Lactobacillus plantarum* ZDY04 significantly reduced serum TMAO and cecal TMA levels by modulating the relative abundance of beneficial *Lachnospiraceae*; also, *L. plantarum* ZDY04 is shown to significantly inhibit the development of TMAO-induced atherosclerosis^28^. Together, these data suggest that the administration of specific strains may possibly increase and/or decrease specific microbial populations in the gut, even though the molecular mechanism by which gut microbiota produce TMA remains unknown.

In patients with ACS, the accurate diagnosis of cardiovascular events by ECG and conventional laboratory tests is challenging and clarification of ACS risk factors is ongoing. Previous study has shown that age, sex, hypertension, and blood cholesterol may be risk factors for ACS patients^29^. In the present study, comparison of ACS patients’ demographic and clinical characteristics, including age, sex, systolic and diastolic blood pressure levels, alanine aminotransferase (ALT), lipoprotein (a), admission glucose, uric acid, white blood (WBC) count, platelet count, albumin and high-density lipoprotein, with those of healthy controls revealed significant differences between the two groups. In order to improve outcomes in patients with cardiovascular heart disease, research has focused on novel risk factors, with special attention given to the interaction between environmental and genetic factors^30,31^. Although nutrition and dietary considerations have long been linked to outcomes in cardiovascular patients, the pivotal role of the gut microbial taxa has only recently been recognized. For instance, elevated TMAO levels have recently been associated with increased mortality risk in patients with chronic heart failure^32^, high risk of incident MACE in those with ACS^33^, and increased all-cause mortality or reinfarction after acute MI^34^. Previous studies have shown that the multi-organismal pathway of TMAO is linked clinically and mechanistically with the development of atherosclerotic plaque, alteration in macrophage and endothelial cell phenotypes, promotion of platelet hyperresponsiveness, and whole-body cholesterol and sterol metabolism^8,9,11,17–19^. In addition, circulating levels of TMAO have been shown in multiple distinct clinical studies to be associated with ACS risk^8,10,14,20,21,25^ and, more recently, targeted suppression of microbial TMA/TMAO production has been shown to inhibit diet-induced atherosclerosis^35^. However, while these investigations have assessed the prognostic implications of TMAO in terms of overall cardiovascular disease risk, no studies to date have directly examined the prognosis of patients with STEMI. In the present study, serum TMAO levels demonstrated an inclination for risk prediction in combination with established clinical information, highlighting the potential clinical application of TMAO as a prognostic risk biomarker for incident MACE and total one-year events in patients with STEMI. In addition, the abundance of TMAO-associated gut microbial taxa, including *Aerococcaceae, Eubacterium*, and *Lachnospiraceae*, was associated with the occurrence of ACS or STEMI, but not with incident MACE post-STEMI. These findings also expand our understanding of the relationship between TMAO and post-STEMI cardiovascular events, suggesting that specific gut microbes may influence the incident cardiovascular events via generation of TMAO.

Clinically, TMAO may be a secondary risk stratification biomarker when used in combination with the Global Registry of Acute Coronary Events (GRACE) score. In patients with higher GRACE scores (>119), TMAO could further define higher risk for 6-months mortality among MI patients, and helped to predict risk in patients with acute MI^34^. Additional stratification using TMAO further defined patients having lower risk as those serum TMAO level was ≦3.7 μM, a value equal to the cohort median and in agreement with healthy reference values, generally <5 μM^9,15,20,36^. Higher plasma TMAO levels in generally stable patients with significant coronary artery stenosis were associated with a four-fold increased mortality risk, and TMAO levels remained predictive of 5-year all-cause mortality risk^37^. However, while previous studies have observed a positive association between systemic TMAO levels and incident cardiovascular risk, certain other studies have not^38,39^; it should be noted that these latter two studies included subjects with impaired kidney function and poor metabolic control, which may have different patterns for TMAO as shown, for example, in diabetes patients^21^. Interestingly, a recent clinical study of the prognostic value of TMAO in subjects on hemodialysis observed that the strength of the association between TMAO and adverse events differed somewhat by race, with whites showing a significant association between higher TMAO levels and heightened incident risk of cardiac death, sudden cardiac death, first cardiovascular event, and any-cause death; in blacks, elevated TMAO levels were significantly associated only with cardiac death^40^. Data of the present study show that serum TMAO levels maintain strong prognostic significance, with higher TMAO levels (>2.19 μM) indicating increased risk of incident cardiovascular events in patients with STEMI.

Mechanistically, a causal association between TMAO and atherosclerosis remains to be established. Atherosclerotic plaque contains microbial DNA, and the microbial taxa are found in the gut of the same individuals^41,42^. These observations suggest that the gut microbial communities may be a source of microbes in the plaque, which may impact plaque stability and CVD development. The gut microbiota of patients with STEMI may thus be fostering inflammation by producing more proinflammatory molecules, leading to more cardiovascular events after STEMI^16^. In addition, patients with increased TMAO levels are at risk for thrombotic diseases, including MI and stroke, suggesting a possible mechanistic link with platelet hyperreactivity, although platelet dysfunction was not found in patients with ischemic events^12^. Recently, a mechanistic link between the gut microbiota and the severity of MI was reported in rats^43,44^. In those studies, the use of broad-spectrum antibiotics was shown to affect levels of leptin and analytes produced during aromatic amino acid catabolism (e.g., phenylalanine, tryptophan, tyrosine), and subsequently reduce myocardial infarct size. Compared to these findings, results of the present study showed that elevated levels of the gut microbe metabolite TMAO, but not IL-6 levels, were associated with the occurrence of ACS and post-STEMI cardiovascular events. However, we cannot infer from the lack of change in IL-6 levels that TMAO does not operate by regulating proinflammatory molecules, since the regulatory effects of IL-6 in inflammation and infection is complicated, and many other pro- and anti-inflammatory cytokines were not measured in this study. Clearly, further investigation is needed to determine whether TMAO influences the occurrence of ACS and post-STEMI cardiovascular events by regulating platelet hyperreactivity or specific proinflammatory molecules.

In rodent studies, administration of *Lactobacillus plantarum* was associated with a significant reduction in infarct size and improved left ventricular function after MI^43^. In addition, the supplementation of *Lactobacillus plantarum* significantly reduced serum TMAO and cecal TMA levels by modulating the relative abundance of the beneficial microbe family *Lachnospiraceae* in mice^29^. Another animal study showed that administration of the *Lactobacillus rhamnosus* GR-1 attenuated left ventricular hypertrophy and heart failure after experimental MI^45^. In recent clinical trials, increased efforts have been applied to reduce the number of cardiovascular events in MI patients. A 36% reduction was seen in all endpoints during 1-year follow-up of MI patients treated with antibiotics in the STAMINA trial^46^. These observations may suggest that probiotics use, in combination with standard medications, may offer additional benefits to patients with ACS, such as reducing the severity of heart failure and the occurrence of cardiovascular events after MI. Although the underlying mechanism has still not been identified and warrants further investigation, evidence of the association between the gut microbiota, TMAO and cardiometabolic disease spurs investigators to continue exploring the gut microbiota as a potential therapeutic target for cardiovascular disease^47^.

## Limitations

The present study has several limitations. First, the sample was relatively small and all included patients were from a single-center in China, suggesting that results may not be generalizable to other populations or geographic areas. Secondly, only a single time-point blood draw after overnight fasting was available, and we did not have a dietary history to confirm or refute the impact of diet on TMAO levels or their cardiorenal consequences. Also, because the blood samples were taken at admission, with no further connection with the patients, we did not have any information about gastrointestinal symptoms and pathology or prior antibiotic use or knowledge of prior supplements other than those taken the day of enrollment. Thirdly, our controls were healthy volunteers rather than patients with coronary atherosclerosis, which is suboptimal since differences between the control group and STEMI and ACS patients may affect interpretation of results; for example, the control group had a lower percentage of males than the ACS group, and sex is a known risk factor for CVD, which may limit the interpretation of findings. We must also note that the controls were not matched for age and gender with the ACS group, although the patients were consecutive and no enrollment bias was found. In general, we found it difficult to enroll patients with coronary atherosclerosis but without ACS/STEMI as a control in this study, because most patients in CCU are ACS, including UAP and STEMI, and very few patients are stable angina pectoris. Finally, the results of this clinical study may suggest a possible trend rather than definitive results and all results should be verified in an animal model or a larger, multi-center trial.

## Conclusions

Results of the present study revealed the presence and abundance of specific gut microbial taxa that may serve as potential predictive biomarkers for accurate diagnosis of ACS onset, including the increased abundance of *Aerococcaceae, Ruminococcaceae_UCG.005, Ruminococcaceae_UCC.014* and *X. Eubacterium_fissicatena*, and the decreased abundance of *Lachnospiraceae_FCS020*. These findings provide novel insight into the link between specific gut microbial taxa-related TMAO formation and the occurrence of ACS and incidence of post-STEMI cardiovascular events. Strategies focused on protecting the gut barrier and reducing or eliminating TMAO-related gut microbes may help to reduce the occurrence of ACS. Further investigation is warranted to evaluate whether targeted interventions can alter specific gut microbial taxa to lower TMAO levels or enhance TMAO clearance. Studies are also needed to determine whether regulating platelet hyperreactivity may alter the occurrence of ACS and post-STEMI cardiovascular events.

## Supplementary information

Supplementary Information.

Supplementary Information 2.
