# Supplementary material for: Gut microbial taxa as potential predictive biomarkers for acute coronary syndrome and post-STEMI cardiovascular events
Source: Sci Rep. 2020 Feb 14;10:2639. doi: 10.1038/s41598-020-59235-5 (PMC7021689; doi:10.1038/s41598-020-59235-5)
Supplement: Supplementary file 1 — Supplementary Information. [file 41598_2020_59235_MOESM1_ESM.docx]

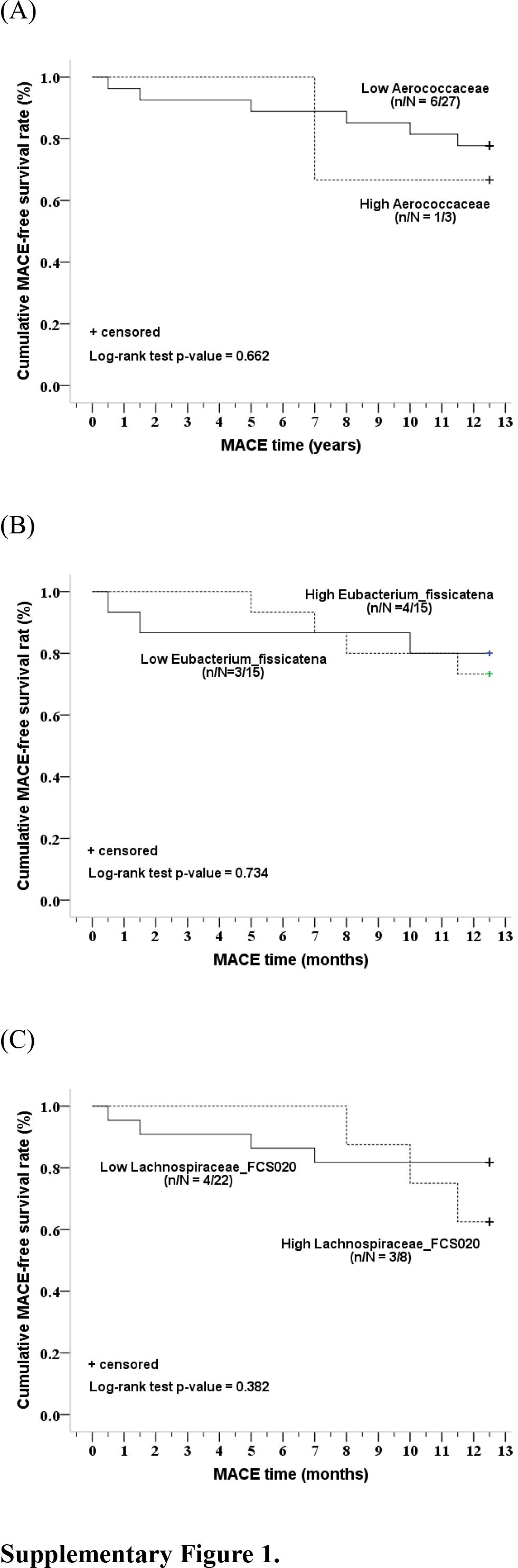


**Supplementary Figure 1.** Comparison of 1-year MACE-free or total event-free survival rates between patients with low and high abundance of (A) *Aerococcaceae*, (B) *Eubacterium_fissicatena*, and (C) *Lachnospiraceae_FCS020*. Patients were stratified according to the mean abundance of microbes = 0.29×10^-5^ for *Aerococcaceae*, mean value = 1.86×10^-5^ for *Eubacterium_fissicatena*, and median abundance of microbes = 0.12×10^-3^ for *Lachnospiraceae_FCS020* in controls. n indicates MACE, N indicates total number of patients. No significant differences were found between microbes.
