# Supplementary material for: Gut microbial taxa as potential predictive biomarkers for acute coronary syndrome and post-STEMI cardiovascular events
Source: Sci Rep. 2020 Feb 14;10:2639. doi: 10.1038/s41598-020-59235-5 (PMC7021689; doi:10.1038/s41598-020-59235-5)
Supplement: Supplementary file 2 — Supplementary Information 2. [file 41598_2020_59235_MOESM2_ESM.docx]

**Gut microbial taxa as potential predictive biomarkers for acute coronary syndrome and post-STEMI cardiovascular events**

Gao J; Yan KT ; Wang JX; Dou J; Wang J, Ren M; Jing Ma J ; Zhang X ; Liu Y

**Supplementary Table 1**. Comparison the demographics and prognostic markers between STEMI patients with and without MACE recurrence. (n=30)

|  | STEMI patients | |  |
| --- | --- | --- | --- |
| Variables | MACE(+) | MACE(-) | p-value |
| *n* | 7 | 23 |  |
| ***Demographics*** |  |  |  |
| Sex, males (%) | 6 (85.7) | 14 (73.7) | 1.000 |
| Age, year | 56 (52 , 62) | 55 (44 , 61) | 0.461 |
| BMI (kg/m^2^) | 22.1 (21 , 25.7) | 26.2 (25.3 , 27.6) | 0.073 |
| HR (/min) | 83.5 (74 , 96) | 74 (65 , 92) | 0.338 |
| SBP (mmHg) | 141 (112.5 , 148.5) | 140.5 (120 , 150) | 0.572 |
| DBP (mmHg) | 82 (75.5 , 93.5) | 88 (70 , 90) | 0.712 |
| WBC (10^9^/L) | 10.8 (10.3 , 14.7) | 10.2 (9.2 , 13.4) | 0.270 |
| HB (g/L) | 130 (110.5 , 155) | 142 (130 , 149) | 0.573 |
| PLT (10^9^/L) | 209 (158 , 286.5) | 238 (203 , 283) | 0.864 |
| ALB (g/L) | 39.3 (33.3 , 41.9) | 41 (38.7 , 41.4) | 0.269 |
| ALT (U/L) | 54 (36.7 , 73.3) | 47.7 (27 , 66.4) | 0.624 |
| Lp(a) (nmol/L) | 59.9 (8.7 , 105) | 23.4 (8.5 , 81.9) | 0.806 |
| TC (mmol/L) | 4.2 (3.5 , 4.6) | 4.6 (4.1 , 5.3) | 0.364 |
| TG (mmol/L) | 1 (1 , 1.2) | 1.8 (1.4 , 2.3) | **0.029*** |
| HDL-C (mmol/L) | 0.9 (0.8 , 1.1) | 1 (0.8 , 1.1) | 0.462 |
| LDL-C (mmol/L) | 3 (2.2 , 3.4) | 2.8 (2.7 , 3.4) | 0.607 |
| GLU (mmol/L) | 6.2 (5.5 , 6.7) | 6.4 (5.5 , 8.3) | 0.825 |
| CR (umol/L) | 76.5 (56 , 123.5) | 66.5 (63 , 86) | 0.229 |
| UA (umol/L) | 344 (188.5 , 390.5) | 363.5 (294 , 435) | 0.750 |
|  |  |  |  |

Data were represented as n(%) for sex and median (1^st^, 3^rd^ quartiles) for other variables; and different between patients with and without MACE were compared using Fisher’s exact test for sex and Mann-Whitney U test for other variables.

^*^indicated significantly (p<0.05).

**Supplementary Table 2**. The effective number of reads, HQ-bases, and sequences.

|  |  | Number of reads | Percentage of HQ bases | |  | Number of Sequences | |
| --- | --- | --- | --- | --- | --- | --- | --- |
| Group | Sample number | Average ± SD | Average ± SD | (Min. to Max) |  | Average ± SD | (Min. to Max.) |
| ACS | 60 | 18583512.20 ± 5299789.82 | 85.13 ± 3.22 | (74.63 to 91.85) |  | 73754.62 ± 21048.06 | (42,293 to 116,291) |
| Control | 25 | 20130814.48 ± 5140216.70 | 89.64 ± 2.14 | (85.44 to 92.76) |  | 79955.04 ± 20375.82 | (45,072 to 111,015) |
| Note: No significant difference derived between ACS and control groups (p>0.05). | | | | | | | |

**Supplementary Table 3.** ROC curve analysis of prognostic

markers, TMAO and IL-6 for identifying MACE event.

| Prognostic markers | AUC (95%CI) | p-value |
| --- | --- | --- |
| TMAO (μM) | 0.729 (0.481 , 0.978) | 0.078 |
| IL-6 (ng/ml) | 0.511 (0.247 , 0.776) | 0.931 |
